# Cuproptosis predicts the risk and clinical outcomes of lung adenocarcinoma

**DOI:** 10.3389/fonc.2022.922332

**Published:** 2022-08-08

**Authors:** Qin Hu, Runtian Wang, Huiyun Ma, Zhouwei Zhang, Qun Xue

**Affiliations:** ^1^ Research Center of Clinical Medicine, Affiliated Hospital of Nantong University, Nantong, China; ^2^ Medical School of Nantong University, Nantong University, Nantong, China; ^3^ Department of Oncology, The First Affiliated Hospital of Nanjing Medical University, Nanjing, China; ^4^ Department of Cardiothoracic Surgery, Affiliated Hospital of Nantong University, Nantong, China

**Keywords:** Copper-induced cell death, cuproptosis, lung cancer, tumor microenvironment, immunotherapy sensitivity, chemotherapy sensitivity, clinical outcomes

## Abstract

Copper is an essential microelement for the body and a necessary coregulator for enzymatic reactions, yet an unbalanced copper level promotes reactive oxidation and cytotoxicity, which ultimately induces cell death. Several small molecules targeting copper-induced cell death have been investigated, yet few showed promising therapeutic effects in clinical trials. In March 2022, *Science* first introduced the concept and mechanisms of cuproptosis, suggesting that copper-induced cell death targets the tricarboxylic acid (TCA) cycle *via* protein lipoylation. Does this novel form of cell death take part in tumorigenesis or tumor progression? Is cuproptosis related to clinical outcomes of diseases? Is there a cuproptosis-related panel for clinical practice in cancer treatment? Herein, based on 942 samples of lung adenocarcinoma (LUAD), we analyzed on gene set level the existence and predictive value of cuproptosis in disease diagnosis and treatment. We screened out and identified the “cupLA” panel which indicates the risk of LUAD occurrence, clinicopathological features of LUAD patients, and could guide clinicians to refine LUAD subtypes and make treatment choices.

## Introduction

Lung cancer is one of the most common causes of cancer-related deaths and is divided into small cell lung cancer and non-small cell lung cancer (NSCLC) according to pathological features. As the most prevalent subtype of NSCLC, lung adenocarcinoma (LUAD) accounts for 40-70% of all NSCLC cases (1). Diagnosis of early NSCLC is mainly based on pathological examination on lung biopsy *via* bronchoscopy, percutaneous needle biopsy and exfoliative cytology (2). Although in recent years, liquid biopsy emerges as a promising minimally invasive diagnostic method (3), requirements for test equipment increase cost and limit the application of this approach. CT scan remains the main technique for follow-up observations for patients with lung cancers, however, due to its limited resolution, regular CT scan fails to monitor the minor progression of the malignancy, such as micro-metastasis, early drug resistance, not to mention long-term prediction of disease outcomes. Therefore, a comprehensive evaluation is of urgent need in clinical practice.

Copper is an essential microelement to the body and a necessary co-regulator of various enzymatic reactions. Copper overloading inside cells is associated with toxicity mediated by reactive oxide species (ROS) in mitochondria. Previously, Golub and colleagues reported cuproptosis as an independent form of cell death in which components of the TCA cycle are lipoylated by copper, revealed dysfunction of copper homeostasis-related genetic models, and suggested that copper homeostasis is a double-edged sword for cellular biology (4). Various research investigated the mechanism of copper-induced cell death. Research on neurotoxicity suggested that copper deregulates the expression of host genes in olfactory signal transduction *via* miRNA-mRNA pathway (5). Copper overloading also contributes to inflammasome activation and damage to nerve cells in the central nervous system (6). Besides, cell death associated with copper toxicity has been researched mainly on metabolic disorders (7). For cells with disorders of copper metabolism, for example, Wilson’s disease (WD), hepatocellular cells activate autophagy as a defensive measure against copper accumulation (8). For cancer cells treated with copper complexes undergoing MAPK pathway activation, oxidative stress as well as eventual cell death (9), and the following ROS-related cell death renders the tumor tissue more immunogenic (10). As a newly revealed form of cell death, the subsequent effects on cancer cells themselves and the surrounding microenvironment are not fully understood. Hepatocellular cells that are exposed to overloading copper accumulation have a higher risk of transformation and carcinogenesis (11). Copper pathways such as the ATOX-ATP7A-LOX pathway facilitate the metastasis of cancer cells, and downregulation of this pathway impedes breast cancer metastasis *in vivo (* 12). For hepatocellular carcinoma (HCC), disulfiram/copper (DSF/Cu) impairs mitochondrial homeostasis, and enhances oxidation-related cell damage, while DSF/Cu synergizes the cytotoxic effects of Raf inhibition (13). The above studies suggested the potential value of copper homeostasis and the subsequent cell death caused by copper overloading in cancer biology, yet most are based on pre-clinical experiments without large-scale research based on clinical samples.

In this research, based on data analysis of high-throughput sequencing, we investigated the expression of cuproptosis biomarkers in 942 LUAD samples, figured out a cuproptosis-based gene model named cupLA (DLD, PDHA1, CDKN2A, DLAT and PDHB) which was correlated with poor clinical outcomes of LUAD patients, and inferred how LUAD cells influenced immune cell infiltration *via* cuproptosis. Overall, we demonstrated the association between a new mode of cell death and the malignancy of LUAD samples, thus indicating the value for further research targeting this cellular mechanism.

## Materials and methods

### Data source and pretreatment

RNA expression and clinical prognosis data were obtained from The Cancer Genome Atlas (TCGA; https://portal.gdc.cancer.gov/) and GSE68465 (https://www.ncbi.nlm.nih.gov/geo/query/acc.cgi) databases. For TCGA dataset, RNA sequencing data was converted from FPKM into transcripts per thousand base million (TPM). The two datasets were combined and the batch effect of non-biotechnology bias was eliminated by using operational algorithms. After excluding the samples with either lack of clinical data or survival data less than or equal to 0, a total of 942 LUAD ones were included for follow-up analysis.

### Somatic CNV and mutation detection

Somatic mutation data of TCGA-LUAD data was downloaded from the Genomic Data Commons (GDC; https://portal.gdc.cancer.gov/), and identified by MuTect2(V4.1.0.0) were sorted in mutation annotation format (MAF) file, and analyzed by R software package(Bioconductor V3.15; https://www.bioconductor.org/) “maftools” (14). The R package “igraph” was used to visualize common frequent mutations shared by different variants, tumor mutation load (TMB) and non-synonymous somatic mutations.

### Construction of CuRGs genotyping

A total of 10 Cuproptosis-related genes (CuRGs) were obtained from the up-to-date paper (4). After survival analysis, the genes with significant survival significance with P value less than 0.05 were selected for consensus unsuper-vised clustering analysis *via* “ConsensusClusterPlus” package (Bioconductor V3.15; https://www.bioconductor.org/) (15). The optimal K value was determined according to the cumulative distribution function and delta area value, and LUAD patients were divided into different clusters according to the differentially expressed CuRGs.

### Functional enrichment analysis

Clinical information (smoking history, race, first treatment outcome, tumor status, residual tumor, stage, grade, TNM, and age) was extracted to plot heatmaps. The gene expression levels were compared by one-way ANOVA. The cluster values of immune cells and immune-related pathways were calculated using the “ssGSEA”(single sample gene set enrichment analysis, ssGSEA) package. The “gsva”(Gene Set Enrichment Analysis,GSEA) package was applied to distinguish the pathways of two clusters. Further, PCA diagrams were drawn using the “STATS” package to describe gene grouping.

### Further typing of differentially expressed CuRGs in the two clusters

In CuRGs, genes with significant differences between the two clusters were selected for further Non-negative matrix factorization (NMF) typing. The methods described above were employed to analyze the clinical relevance of the newly clustered genes.

### Construction and validation of a prognostic signature

Using the “caret” package, 942 samples were divided into training and verification groups in the ratio of 1:1, in which the output conditions was [(pvalue < 0.01) & (ROC $AUC [2] > 0.65) & (pvaluetest < 0.05) & (ROCTEST $AUC [2] > 0.63)]. Furthermore, based on the five CuRGs, significantly expressed in two different NMF subtypes, lasso regression algorithms were used to minimize the risk of overfitting using the “glmnet” R software package. The changing trajectory of each independent variable was analyzed and the characteristics were constructed using 10-fold cross-validation. Finally, multivariate Cox analysis was applied to select candidate genes and construct the risk signature in the training set.Risk characteristics are defined as follows: Risk score = ∑ (EXPI) × Coefi).

Survival software package (Version 3.2-10) was used to narrow down candidate genes and develop prognostic models. Ggplot2 software package(Bioconductor V3.15; https://www.bioconductor.org/) was used for risk factor graph analysis, and timeroc (Version 0.4) package was used for ROC curve analysis. Univariate and multivariate Cox regression models, Kaplan-Meier method and bilateral Log rank test were used to compare patients’ OS between subgroups and evaluate the independent prognostic value of risk models. The “survConcordance” package was employed to calculate the time-dependent consistency index (C-index) to analyze and compare the survival prediction ability between different variables.

### Construction of a nomogram for LUAD patients

To quantify the risk assessment of individual patients with LUAD, based on “rms” package, a personalized score Nomogram was generated to predict the 3-year and 5-year progression-free survival probabilities of patients using these four parameters. The calibration diagram shows that the line diagram operates in accordance with the ideal model.

### Evaluation of characteristics of infiltrating immune cells and immune microenvironment

ssGSEA and MCPcounter were calculated to evaluate the activity of immune-related pathways by infiltrating immune cell scores. To investigate the relationship with risk characteristics, Estimated STromal and Immune cells in MAlignant Tumour tissues using Expression data (ESTIMATE) score was used to calculate the estimated score of the tumor microenvironment and the immune score of the tumor. In the analysis of immunotherapy in the different groups of the signature, we made use of Tumor Immune Dysfunction and Exclusion (TIDE; http://tide.dfci.harvard.edu/login/) and The Cancer Immune Database (TCIA; https://tcia.at/home) (16, 17). In addition, we analyzed the correlation between model genes and immune checkpoints with “reshape2” package. Ultimately, the sensitivity of the drug to the high - and low-risk groups was shown using a bar chart. All statistical analyses were performed using R software (Version4.1.2) (18).

## Results

### Identification of cuproptosis biomarker genes in lung adenocarcinoma

**Figure 1:**
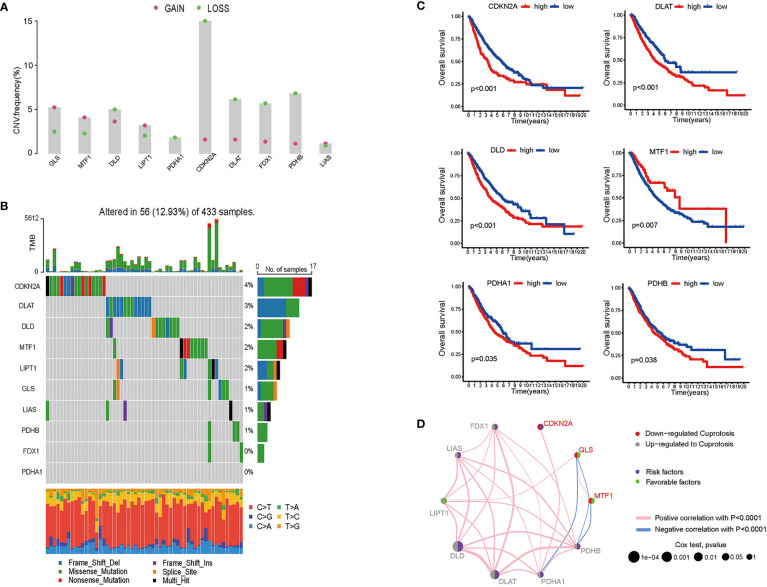
Genomics and transcriptomics-based bioinformatic analysis on cuproptosis-related genes in lung adenocarcinoma. **(A)** CNV frequencies showed the loss and gain of ten cuproptosis biomarkers. **(B)** TMB of cuproptosis biomarkers and the proportion of base-pair alterations in LUAD samples. (For exmale, C>T: cytidine being replaced by adenosine, and so on in a similar fashion.) **(C)** ROC curves showed overall survival of single cuproptosis-related gene expression in LUAD patients. **(D)** Association between cuproptosis biomarkers expression and LUAD risks. (For example, DLD was up-regulated in LUAD samples; DLD expression was a risk factor for LUAD patients; and DLD expression was positively correlated with LIPT1, LIAS, FDX1, PDHB, PDHA1, and DLAT).

For 490 LUAD samples from the TCGA database, we identified the expression of cuproptosis biomarkers represented by GLS, MTF1, DLD, LIPT1, PDHA1, CDKN2A, DLAT, PDX1, PDHB and LIAS (4). Since copy number variant (CNV) results in amplification or deletion of gene expression, investigating CNV characteristics of cancer-related genes helps to understand tumorigenesis and explore potential targets for cancer treatment (19). To probe the clinical significance of cuproptosis in LUAD, we inferred the CNV of ten cuproptosis biomarkers based on transcriptome data (**Figure 1A** and **Figure S1**). Among the LUAD samples from TCGA database, the majority of patients gained amplification of GLS, MTF, LIPT1 and LIAS, and depletion of DLD, PDHA1, CDKN2A, DLAT, FDX1 and PDHB. GLS encodes glutaminase which functions as a substrate of MET kinase and energizes tumor migration *via* the Warburg effect (20). MTF is a pro-tumoral gene promoting the proliferation of melanoma cells, and GLS is one of the differentiated expressed genes of MTF silencing tumors (21). LIPT1 functions as an activator in the TCA cycle by 2-ketoacid dehydrogenases (22). LIAS encodes lipoic acid synthase whose mutation stabilizes the HIFα signaling pathway, thus mediating adaptation to a hypoxic environment (23). DLD takes part in stabilizing the biological activity of mitochondria and is downregulated by UVA, leading to NAD+/NADH imbalance and cell death in melanoma (24, 25). The PDH family constitutes pyruvate dehydrogenase complex and modulates cancer cells in oxidative stress through TCA cycle (26), and DLAT encodes the E2 subunit of mitochondrial PDH complex (27). CDKN2A is a common inactivated gene across cancer types, and it inhibits cancer cell proliferation by targeting the cell cycle (28). FDX1 also takes part in energy consumption mediated by nutrients (29). 12.93% were detected to have CNV alteration related to cuproptosis, with missense and deletion mutation accounting for the majority (**Figure 1B**). Primary analysis indicated the potential value of studying cuproptosis in lung adenocarcinoma, therefore, for better comprehensiveness and consolidation, we included GSE68465 which included transcriptome profiles of 442 lung adenocarcinomas (30). After batch effects removal, TCGA samples and GSE68465 samples were incorporated, increasing the sample size to 942 for further research.

To determine whether cuproptosis is associated with clinical outcomes of LUAD patients, we plotted the overall survival (OS) curves for the ten cuproptosis biomarkers (**Figures 1C, D**). Among the ten genes modulating cuproptosis, DLD, PDHA1, CDKN2A, DLAT and PDHB were significantly correlated with poor OS in a 10-year term, while MTF1 expression indicates longer OS. This finding is distinct from a study on ovarian cancer where the transcription factor MTF1 promoted the epithelial-mesenchymal transition (EMT) *via* genomic mechanisms and intercellular interactions, thus impeding the migration and metastasis of cancer cells (31). The OS curves primarily screened out six genes statistically associated with the prognosis of lung adenocarcinoma, with five predicting poor overall survival and one possibly being a favorable factor.

### The establishment of a cuproptosis model in lung adenocarcinoma

**Figure 2:**
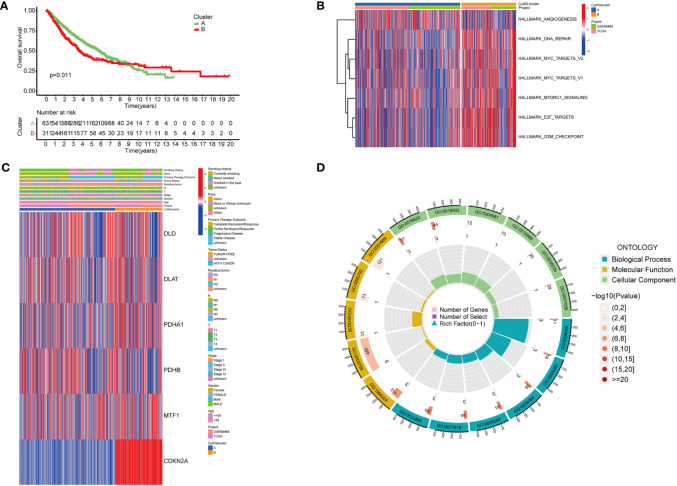
Preliminary screening of cuproptosis-related genes indicative of LUAD survival. **(A)** Kaplan-Meier curves for OS of two LUAD subgroups automatically clustered by transcriptomic profiles. **(B)** Enrichment analysis showing characteristics of two LUAD subgroups. Cluster B with poorer five year-survival are enriched in pathways including DNA repair, MYC targets, MTORC1 signaling, E2F targets, and G2M checkpoints. **(C)** Expression of six cuproptosis biomarkers related to poor overall survival and their relationships with clinicopathological features. **(D)** GO enrichment analysis of the six-cuproptosis marker genes.

To better investigate whether the cuproptosis-related gene set can be used as predictive biomarkers for clinical outcomes of LUAD patients, we performed unsupervised clustering and classified all patients into two subgroups (**Figure S2**). Further analysis of clinical outcomes demonstrated that the OS of subgroup B was shorter than subgroup A (**Figure 2A**). Based on the differential transcriptomics, we figured out the top enriched pathways of both subgroups (**Figure 2B**). Subgroup B was significantly more active in pathways that facilitate tumor progressions, such as angiogenesis, MYC-mediated targets, and the mTOR signaling pathway. Besides, cell cycle and cancer cell proliferation pathways such as DNA repair, E2F targets, and cell cycle checkpoints are also highly enriched according to the differentiated transcriptome. Notably, when interrogating LUAD samples on the expression of the six-gene cuproptosis model, we found universally high expression of CDKN2A in subgroup B, while the moderate inclination of either subgroup with the other five genes (**Figure 2C**). Previously, we identified six cuproptosis biomarkers that indicate poor clinical outcomes (DLD, PDHA1, CDKN2A, DLAT, PDHB and MTF1). All six genes were expressed in both LUAD subgroups, with a significant differential expression of DLD, PDHA1, CDKN2A, DLAT, and MTF1 in subgroup B. Since this five-gene model showed significant correlations with poor clinical outcomes in lung adenocarcinoma, we name it cupLA panel for further analysis. To better clarify the biological function of the cupLA panel, we performed functional enrichment to identify that this five-gene panel is most associated with the biosynthesis and metabolism of acetyl-CoA, and the composition of mitochondrial matrix (**Figure 2D**). This finding is in accord with the view that intercellular copper trafficking participates in enzymatic redox reactions (32, 33).

### Cuproptosis model in evaluation of LUAD risk

**Figure 3:**
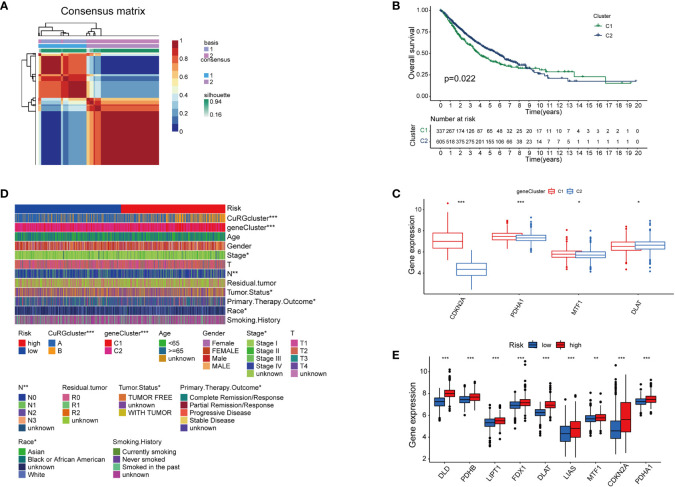
The establishment of cupLA panel and the risk assessment potency. **(A)** Consensus matrix of two LUAD clusters based on cuproptosis-related gene set demonstrated in **Figure 2**. Based on the sis-gene model, two new LUAD clusters (C1 and C2) were identified. **(B)** Kaplan-Meier curves for OS of two C1 and C2. **(C)** Expression of cuproptosis biomarkers in two LUAD clusters. **(D)** Heatmap showing clinicopathological features of patients from the high and low-risk groups and the correspondence with the cuproptosis-based gene set. **(E)** Expression of cuproptosis biomarkers in the high and low-risk group (*, p<0.05; **, p<0.01' ***, p<0.001).

With a preliminary understanding of cuproptosis-related genes in LUAD clinical outcomes, we narrowed the panel down to five differentially expressed genes for further study and named it cupLA panel. Next, we examined the expression of cupLA in all LUAD samples, and clustered samples again based on cupLA expression (**Figure 3A**). Overall survival of two subclusters (C1 and C2) demonstrated that cupLA could serve as a bioinformatic tool with prognostic value in clinical practice (**Figure 3B**). At the gene expression level, C1 has statistically higher expressions of CDKN2A, PDHA1, and MTF1, and a lower expression of DLAT (**Figure 3C**).

To predict the correlation between the cupLA panel and the risk of lung adenocarcinoma occurrence, we performed Lasso regression which therefore identifies LUAD patients with high and low risks (**Figure S3**). Deconvolution analysis suggested the feasibility of our cupLA panel and identified the clinicopathological features of patients with high and low risk of lung adenocarcinoma (**Figure 3D**). We interrogated the expression of ten canonical cuproptosis biomarkers and found that the high-risk group showed general active cuproptosis than the low-risk group (**Figure 3E**). Moreover, the high-risk group has a high expression of the cupLA panel, suggesting that cupLA panel is useful to evaluation for LUAD risk.

### Prognostic value of cuproptosis for LUAD patients

**Figure 4:**
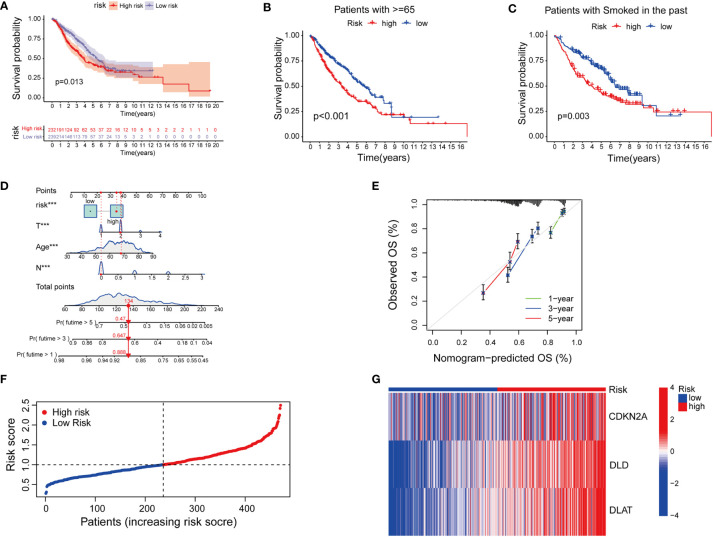
Cuproptosis-related gene model predicts the clinical outcomes of LUAD patients. **(A)** Kaplan-Meier curves of LUAD subgroups divided by cuproptosis-related gene model. **(B, C)** Cuproptosis-related gene model predictive of poor overall survival among patients older than 65 years and smoking history. **(D)** Nomogram showing the efficacies of cuproptosis-related gene model for predicting the RFS of 1, 3, and 5 years in LUAD patients. **(E)** Calibration curves of the nomogram for predicting 1-, 3-, and 5-year OS in LUAD samples. **(F, G)** A risk prediction model based on the cuproptosis-related gene set.

Next, we tried to explore the clinical value of cupLA panels for patients who had already developed lung adenocarcinoma. To determine the potential value of cuproptosis evaluation in clinical prognosis, we studied the correlation between cupLA panel and the clinicopathological features of LUAD patients in TCGA database. Patients in the high-risk group evaluated by cupLA expression showed poor five-, and ten-year overall survival (**Figure 4A**). This panel is particularly predictive for patients over 65 years and with a smoking history (**Figures 4B, C**). For a comprehensive understanding of how cuproptosis is associated with clinicopathological features and to gain a convenient application of the cupLA model in clinical practice, we plotted the Cox test-based nomogram for predicting the risk of lung adenocarcinoma, and information of patients such as age, and indicators of disease severity such as lymph node status (**Figures 4D, E**). Nomogram analysis shows that expression of cupLA panel indicates a high risk of medical history, greater pathological stage, positive lymph nodes, severe tumor status and shorter overall survival (**Figures 4D, E**). The above findings verified that cuproptosis level serves as a predictive biomarker for lung adenocarcinoma, not only the possibility of disease occurrence but also severity of disease progression and poor overall survival. We further identified the value of cupLA in risk prediction in lung adenocarcinoma (**Figure 4F**). Notably, according to Cox regression and nomogram, high-risk LUAD cases have high expression of CDKN2A, DLD and DLAT (**Figure 4G**), also indicating the utility of the cupLA panel in LUAD patients.

### Cuproptosis-related risk and anti-tumoral immune response

**Figure 5:**
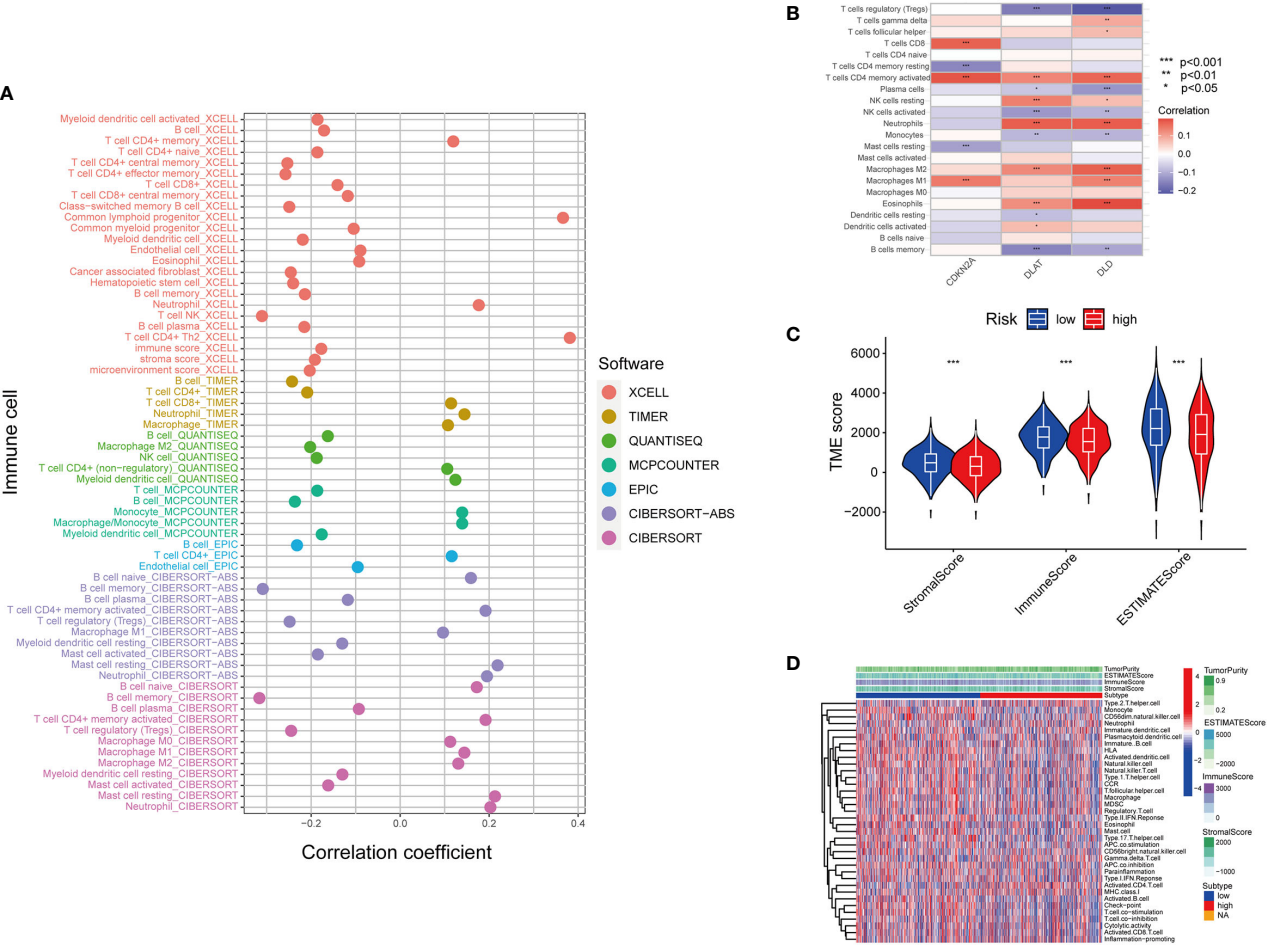
Inferring the TME of LUAD samples with different cuproptosis statuses. **(A)** Correlation between cuproptosis gene model and immune cell infiltration inferred by different bioinformatic tools. (XCELL, TIMER, QUANTISEQ, MCPCOUNTER, EPIC, CIBERSORT-ABS, and CIBERSORT.) **(B)** Correlation between the immune cell infiltration and the differentially expressed cuproptosis-related gene model. (For example, one of the cuproptosis biomarker CDKN2A has a positive correlation with CD8+T cell infiltration, CD4+T cell memory and M1 subtype macrophages.) **(C)** Correlation between cuproptosis-based risk assessment and TME components. **(D)** Estimation of TME components and enriched pathways in the high and low-risk groups determined by cuproptosis-related gene model (*, p<0.05; **, p<0.01' ***, p<0.001.

The tumor microenvironment (TME) is defined as the ecosystem surrounding tumor cells. Composed of cellular and molecular components, TME facilitates the growth of tumor cells and metastatic dissemination (34). Therefore, we utilized several platforms to demonstrate the correlation between the risk score by cupLA panel and TME components (**Figure 5A**). Based on seven bioinformatics tools, we identified that high cupLA expression is positively related to inflammation and anti-tumoral components such as neutrophils, M1 macrophage, CD8+T cells, and macrophage/monocytes, while negatively related to B cell memory, the pro-tumoral M2 macrophage, and myeloid dendritic cells. Since CDKN2A, DLAT, and DLD were the three main biomarkers in predicting cuproptosis-related LUAD risk prediction, we evaluated the expression of these three genes and immune cell abundance in LUAD samples, suggesting that cuproptosis-based LUAD risk prediction suggested more CD4+ memory T cells activation, macrophage abundance and Th cells (**Figure 5B**). Besides, we demonstrated the correlation between cuproptosis-based LUAD risk and TME components (**Figures 5C, D**). Bioinformatics analysis demonstrated that expression of cupLA panel is negatively correlated with immune cell infiltration, suggesting that the transcriptome of tumor tissue could serve as an indicator of anti-tumoral immune response.

**Figure 6:**
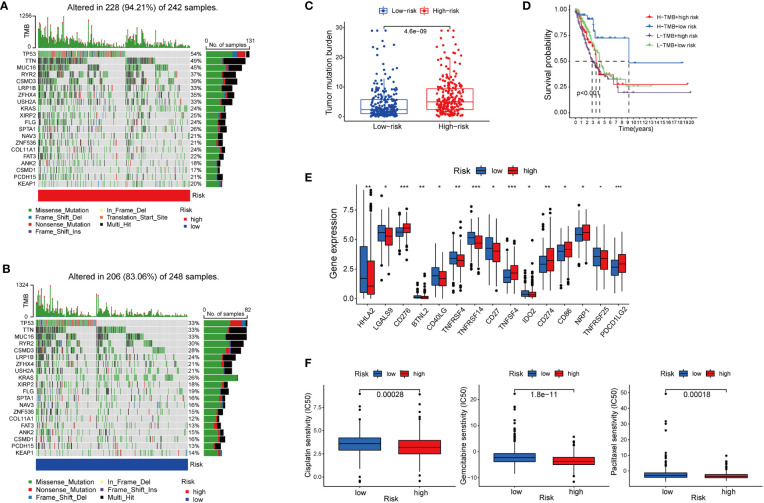
The association between cuproptosis status and sensitivities to immunotherapy and chemotherapy. **(A, B)** Mutations of tumor-associated genes in the high- **(A)** and low-risk **(B)** groups. **(C)** TMB of the high- and low-risk LUAD group. **(D)** Kaplan-Meier curves show the overall survival of patients with different TMB statuses and cuproptosis-based LUAD risks. **(E)** Expression of immune checkpoints in the high and low-risk groups. **(F)** Sensitivities to cisplatin, gemcitabine, and paclitaxel of the high- and low-risk group identified by cuproptosis statuses (*, p<0.05; **, p<0.01' ***, p<0.001.

Besides immune cell infiltration, TMB is another element closely associated with the anti-tumoral immune response. High TMB gives the cancer cells more neoantigens, and renders them more vulnerable to being attacked by effector cells such as CD8+T cells (35, 36). Based on the high-risk and low-risk group divided by the cupLA panel, we identified higher TMB in the high-risk group than in the low-risk group, suggesting a potential correlation between cuproptosis and tumor mutation in lung cancer cells (**Figures 6A–C**). The previous hypothesis indicated that TMB could predict the cancer cell sensitivity to immune checkpoint blockade (ICB), however, a more recent analysis fails to validate the hypothesis, indicating that TMB alone does not predict good clinical outcomes (36). Herein, we combine cupLA panel with TMB to analyze the overall survival of LUAD samples (**Figure 6D**). We find that patients with high-TMB and low risk determined by cupLA panel have the longest survival, while patients with low-TMB and high risk determined by cupLA panel have the shortest survival. To figure out the molecular mechanisms underlying this observation, we interrogate the two groups for expressions of immune checkpoints (**Figure 6E**). CupLA panel demonstrated the high-risk group of lung adenocarcinoma patients, and is positively associated with several canonical molecules related to immune recognition. Among them, co-inhibitory molecules such as CD274 (which is also known as PD-L1) and CD276 present significantly high expression in the high-group samples.

### Cuproptosis-related LUAD risk and chemotherapy sensitivity

Chemotherapy remains the cornerstone of cancer treatment, yet it is unknown which subgroup benefits from chemotherapy better (37). Thus, to test whether cuproptosis is indicative to therapeutic efficacy, we analyzed the drug sensitivity of three commonly used chemotherapy cisplatin, gemcitabine, and paclitaxel based on cupLA panel (**Figure 6F**). Based on the risk evaluation using the cupLA panel, we identified that in lung adenocarcinoma, cuproptosis not only promotes immune evasion, but also renders cancer cells less sensitive to cisplatin, gemcitabine, and paclitaxel. The above analysis demonstrated that based on cuproptosis biomarkers, our cupLA panel predicts the clinical outcomes of LUAD patients, indicates the activity of immune response in tumor tissues and estimates the drug efficacies of commonly used chemotherapies.

## Discussion

Copper is a microelement whose homeostasis is essential for various enzymatic reactions and the proper functioning of organs and metabolic processes, however, previous reports found that copper excess and aggregation cause oxidative stress and cytotoxicity (38). Multiple studies showed that unbalanced copper homeostasis affects tumor growth by interfering with apoptosis, autophagy, oxidative reaction, and angiogenesis (39, 40). And some studies indicated that cupreous ion chelating agents such as elesclomol caused cell death by transporting copper into the mitochondria where reactive oxide species (ROS) were synthesized and accumulated (41). Another research on colorectal cancer revealed that elesclomol also indirectly contributes to intracellular copper aggregation and induced cell death by degrades the copper transporter ATP7A (42). In March 2022, *Science* published an article introducing a novel form of cell death named cuproptosis, which is different from the previously published apoptosis, ferroptosis, pyroptosis and necroptosis (4). Meanwhile, the report shows that copper-induced cell death is mainly accomplished by protein lypoylation of the tricarboxylic acid (TCA) cycle. Moreover, researchers identified seven genes conferring resistance to cuproptosis (FDX1, LIAS, LIPT1, DLD, DLAT, PHDA1, PHDB) and three genes facilitating cuproptosis (MTF1, GLS, CDKN2A) of cuproptosis were identified. Cancer cells undergo metabolic reprogramming and gene mutation that adapt them to the nutrient-deprived, acidic and oxidative microenvironment (43). Since an unbalanced copper metabolism caused cell death, therefore, we wanted to know whether this biological process engaged in cancer cell survival and played a role in cancer progression and could serve as a potential target for cancer treatment.

In this research, samples of lung adenocarcinoma were interrogated with cuproptosis-related genes, among which high expression of DLD, DLAT, PHDA1, PHDB, and CDKN2A were correlated with poor OS, while high expression of MTF1 was correlated with longer OS compared with the MTF1 low expression. As to gene mutation analysis, we found that CDKN2A is the gene with the most mutation identified in TCGA LUAD samples. To enlarge the sample size and increase data reliability, we included transcriptomic information from 942 lung adenocarcinoma samples. Samples were clustered into two by differentially expressed genes, while five cuproptosis-related genes were expressed in both LUAD clusters: DLD, DLAT, PHDA1, MTF1, and CDKN2A. To explore the biological function of these five cuproptosis-related genes, we performed enrichment analysis and found that in lung adenocarcinoma, cuproptosis was most related to the mitochondrial respiration chain. This analysis matches the previous findings that copper-induced cell death targets TCA cycle (4). For a more accurate analysis based on cuproptosis, we narrowed the gene sets for filtering down to the above five genes, and clustered the 942 LUAD samples into Cluster1 (C1) and Cluster2 (C2) based on the expression of the five cuproptosis-related expressions. Patients in C1 have a significantly longer OS than those in C2, and by studying the differentially expressed genes, we screened out four cuproptosis-related genes: CDKN2A, PHDA1, MTF1, and DLAT, naming the four-gene model cupLA panel. By then, we identified a four-gene model, the cupLA panel, which is correlated with poor clinical outcomes in LUAD patients. Therefore, we tried to determine whether cuproptosis indicates the prognosis of lung adenocarcinoma. By integrating clinicopathological features with transcriptome characteristics, we recognized patients with high and low risk of lung adenocarcinoma. Notably, the high-risk group presented overexpression of three cuproptosis biomarkers: CDKN2A, DLD, and DLAT; moreover, tumor tissues of this group have more tumor mutation burden and less immune cell infiltration. As to treatment strategies, the high-risk group has higher expression of co-inhibitory factors and is more resistant to common chemotherapy including cisplatin, gemcitabine, and paclitaxel. The risk assessment demonstrated that on the gene set level, the cuproptosis-related gene set indicates higher disease risk, and more potential to perform immune evasion and establish chemotherapy resistance. Although this research is mainly based on bioinformatics analysis, this is an innovative and cutting-edge study based on big data and could serve as assistance to clinical practice.

Research has unraveled several modes of cell death such as apoptosis, necroptosis, pyroptosis, autophagy, ferroptosis, and cuproptosis. Apoptosis, necroptosis, and pyroptosis cause cell membrane instability and cell rupture by different cellular and molecular mechanisms, such as inflammatory caspases, or ionic gradients (44). Autophagy leads to organelles degradation which provides metabolites, suppresses DNA damage and resists oxidative stress (45). Ferroptosis is driven by phospholipid peroxidation and breaks the REDOX balance (46). Each mode of cell death not only represents a unique mechanism, but also demonstrates immune response under different conditions, moreover, crosstalk exited as well (44, 47). The significance of these mechanisms lies in discovering novel targets with promising efficacy and feasible application. Cuproptosis describes a new mode of cell death resulting from copper overlading and subsequent damage to the TCA pathways located in the mitochondria (48). By studying its correlation to cancer, we had a preliminary understanding of the significance to study this new mode of cell death; by analyzing the molecular mechanisms in cancer progression, we screened out treating targets and prognostic panels; and by utilizing large-scale data from high-throughput sequencing, we gained efficiency to accomplish our goals.

## Conclusions

Taken together, based on gene sets of copper-induced cell death, this study suggested that cuproptosis-based transcriptomic characteristics are indicative to the risk and progression of lung adenocarcinoma. Moreover, for LUAD patients, gene set analysis on cuproptosis biomarkers reflects the TME status and is indicative of drug sensitivity of both chemotherapy and immune checkpoint inhibitors. This is the first large-sample size analysis to reveal the clinical value of cuproptosis in research and clinical practice on lung adenocarcinoma.

## Data availability statement

The original contributions presented in the study are included in the article/**Supplementary Material**. Further inquiries can be directed to the corresponding author.

## Author contributions

QH conceived and designed the study, obtained funding, and drafted the manuscript. QH and RW acquired the data and drafted the manuscript. QH, RW and HM critically revised the manuscript. ZZ and QX performed the statistical analysis and technical support. All authors contributed to the article and approved the submitted version.

## Funding

This work has been funded with support from the Research Center of Clinical Medicine of Affiliated Hospital of Nantong University, Nantong, China. The funders had no role in the study design, data acquisition, data interpretation, or writing of the manuscript.

## Conflict of interest

The authors declare that the research was conducted in the absence of any commercial or financial relationships that could be construed as a potential conflict of interest.

## Publisher’s note

All claims expressed in this article are solely those of the authors and do not necessarily represent those of their affiliated organizations, or those of the publisher, the editors and the reviewers. Any product that may be evaluated in this article, or claim that may be made by its manufacturer, is not guaranteed or endorsed by the publisher.

## Supplementary material

Supplementary Figure 1 Circos plot illustrating regions of ten cuproptosis biomarkers on genome-wide CNV alteration (demonstrating CNV gains of GLS, MTF, LIPT1, and LIAS, and depletion of DLD, PDHA1, CDKN2A, DLAT, FDX1 and PDHB).

Supplementary Figure 2 Construction of LUAD clusters by cuproptosis biomarkers. **(A)** Consensus clustering cumulative distribution function (CDF) identifies two LUAD clusters. **(B)** Consensus clustering matrix of k = 2.

Supplementary Figure 3 Filtering prognostic genes of cuproptosis in lung adenocarcinoma. **(A,B)** LASSO regression analysis of 5, 4, 3, and 2 variables, and the partial likelihood deviance on the prognostic model.

## Abbreviations

TCA, Tricarboxylic acid; LUAD, Lung adenocarcinoma; NSCLC, Non-small cell lung cancer; ROS, Reactive oxide species; WD, Wilson’s disease; HCC, Hepatocellular carcinoma; DSF/Cu, Disulfiram/Copper; TME, Tumor microenvironment; CNV, Copy number variant; OS, Overall survival; EMT, Epithelial-mesenchymal transition; ICB, Immune checkpoint blockade; TCGA, The Cancer Genome Atlas; TPM, Thousand base million; GDC, Genomic Data Commons; MAF, Mutation annotation format; TMB, Tumor mutation load; CuRGs, Cuproptosis-related genes; NMF, Non-negative matrix factorization; TIDE, Tumor Immune Dysfunction and Exclusion; TCIA, The Cancer Immune Database; CDF, Cumulative distribution function.
