# Supplementary figures and images for: Cuproptosis predicts the risk and clinical outcomes of lung adenocarcinoma
Source: Front Oncol. 2022 Aug 8;12:922332. doi: 10.3389/fonc.2022.922332 (PMC9393616; doi:10.3389/fonc.2022.922332)

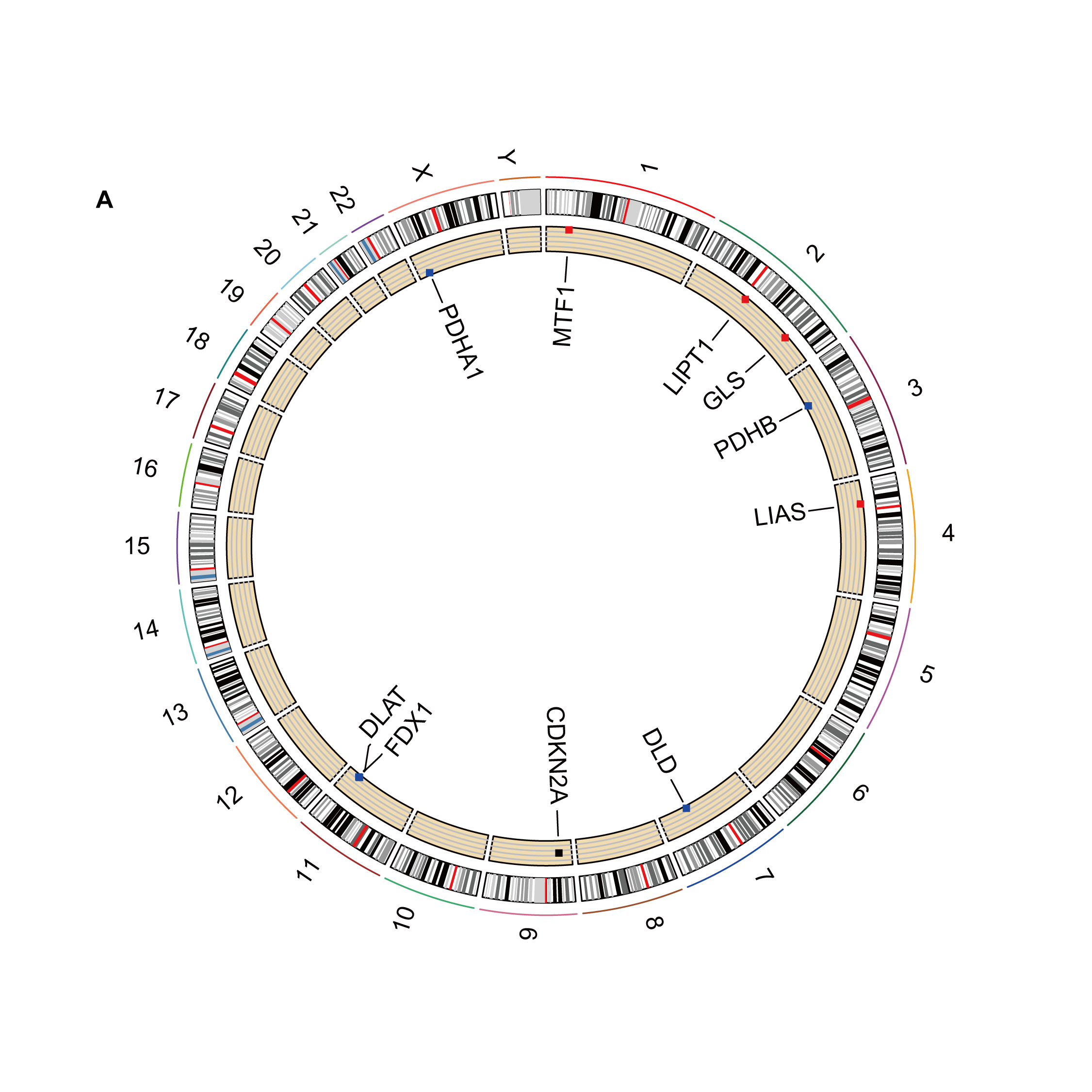

Supplement: Supplementary Figure 1 — Circos plot illustrating regions of ten cuproptosis biomarkers on genome-wide CNV alteration (demonstrating CNV gains of GLS, MTF, LIPT1, and LIAS, and depletion of DLD, PDHA1, CDKN2A, DLAT, FDX1 and PDHB). [file Image_1.tif]

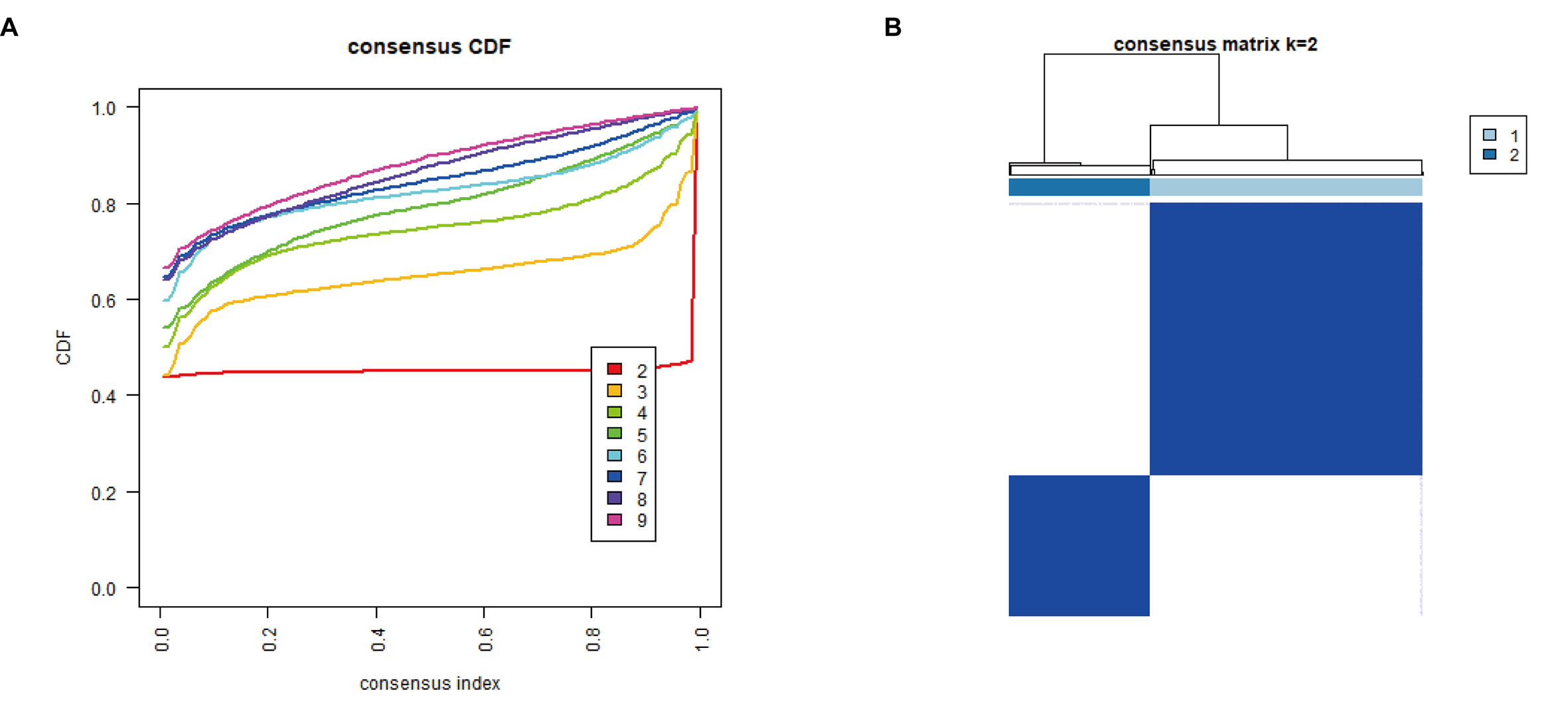

Supplement: Supplementary Figure 2 — Construction of LUAD clusters by cuproptosis biomarkers. (A) Consensus clustering cumulative distribution function (CDF) identifies two LUAD clusters. (B) Consensus clustering matrix of k = 2. [file Image_2.tif]

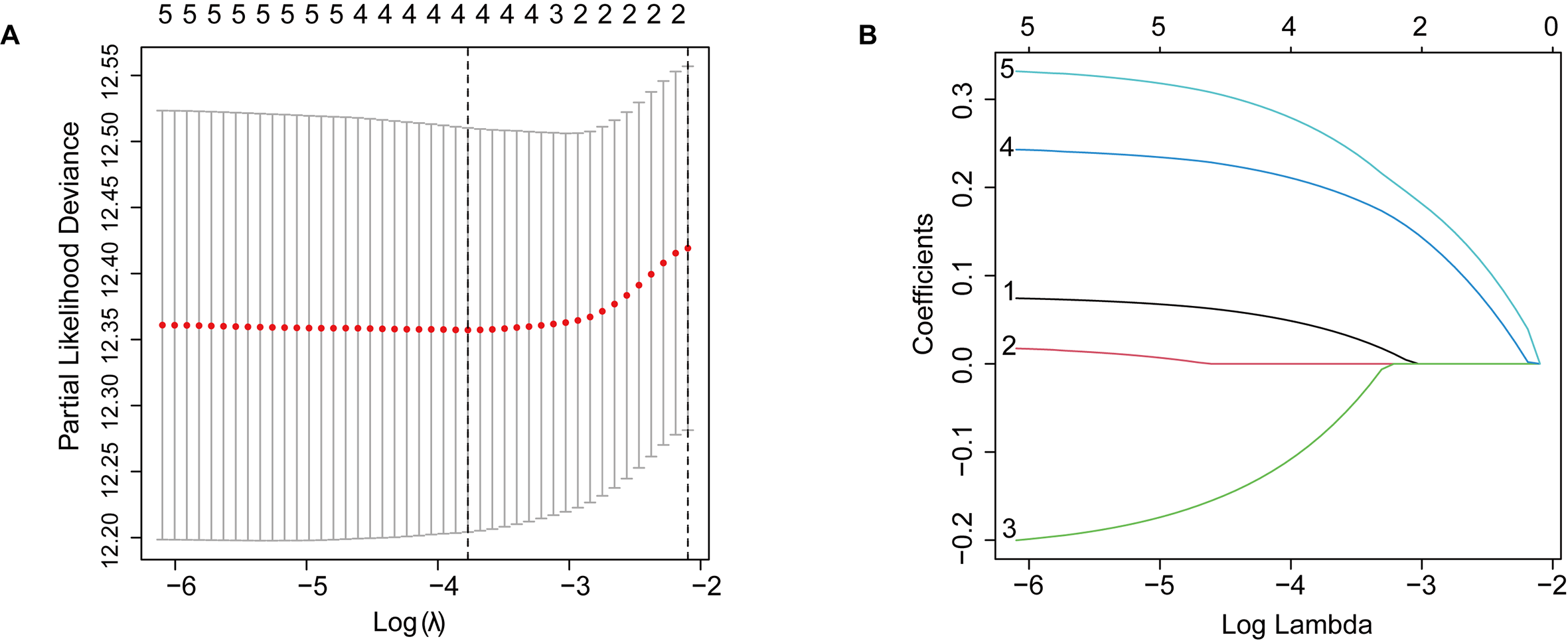

Supplement: Supplementary Figure 3 — Filtering prognostic genes of cuproptosis in lung adenocarcinoma. (A,B) LASSO regression analysis of 5, 4, 3, and 2 variables, and the partial likelihood deviance on the prognostic model. [file Image_3.tif]
